# Notes

**Published:** 1884-05

**Authors:** 


TESTIS IN PERINEO.—Dr. R. L. Macdonnell, in *Canada Medical Record*, August, 1883, relates a case. The patient is fifteen years old. The left testicle has rested in the perineum from the time of his birth. It is situated slightly to the left of the anoscrotal raphe, rather nearer the anus than the scrotum. The organ is well developed, and freely movable. It can be put into its proper place, but cannot be retained there The scrotum is not so well developed on the left side as upon the right. There is left inguinal congenital hernia. The boy has been under observation for the last five years. He is said to have been born prematurely at the sixth month, and up to the present time has been very delicate, but the deformity has, as yet, caused him no inconvenience.—*American Practitioner.*

HOW TO TAKE A PILL.—Dr. Samuel E. Wills, Earlville, Md., suggests the following method:

Having noticed that if a person at meals inclined the head backwards, as in laughing, while there was food in the mouth, they were pretty certain to be strangled from "the food going the wrong way," I instructed those of my patients who had difficulty to swallow pills, to *keep* the head in the position they would if eating and swallowing food at the table—that is, the head inclined forward, the chin near the breast—and *keep* it in that position. If a small portion of saliva be on hand, or a small quantity of water taken after the pill is put in the mouth, it will surprise the patient and gratify the doctor to witness the facility with which it will be swallowed. To direct the patient to keep his eyes on his toes, I have found a help to keep the head in the proper position.—*Med. and Sur. Reporter.*

RULE FOR REDUCING DISLOCATIONS OF THE HIP-JOINT.—Having flexed the leg on the thigh, and the thigh on the pelvis, slowly rotate the limb as far as possible, inward or outward, according as the toes pointed in or out, before beginning the manipulation, then rapidly and forcibly rotate the limb in the opposite direction, and the head of the femur will usually slip into the acetabulum.

For example: In the iliac and sciatic dislocations, the toes point inward; therefore, rotate inward as far as possible, and afterward rotate outward. In the pubic and thyroid dislocations the toes point outward, hence rotate the limb outward still more, and hen inward.—*Polyclinic.*

TUBAL FŒTATION. Dr. E. P. Christian, of Wyandotte, Mich., reports a case of tubal fœtation in a patient forty years old. When he saw the case first, she was suffering with distress and pains of a decidedly paroxysmal character in the lower part of the abdomen, referred by her to the uterus. She could give no definite account of deranged uterine functions. The menses had been as usual.

The patient was concluded to be suffering from an ovaritis, and appropriate treatment prescribed and rest enjoined.

Two weeks later she had well developed peritonitis, and died four weeks later from rupture of the sack. Post-mortem examination revealed a fœtus of 10 weeks' development with feet protruding from and the head still retained in the rent in the sack formed by dilation of the left tube, the forming placenta lining the bottom of the sack. The opening of the containing tube within the uterus was very narrow, not admitting the smallest probe. The uterus was enlarged to about four inches in length, cavity correspondingly enlarged, lining membrane rugose, and slightly injected.—*Detroit Lancet.*

FACIAL ERYSIPELAS.—Prof. Robert Bartholow, in the course of a clinical lecture in Jefferson Medical College on this subject, has this to say of the treatment: In an ordinary case, it will suffice to place the patient at rest, order a suitable diet and keep the bowels open; but if the case is more serious, there are three remedies which may be used with advantage. The first is belladonna. This drug produces a condition of the skin and vessels directly in antagonism to that which exists in erysipelas. You will often be surprised to see how speedily the erysipelas disappears after the development of dry mouth, dilated pupil and flushing of the skin.

If there be any systemic depression, as there usually is in severe cases of facial erysipelas, quinine should be combined with the belladonna giving one-quarter of a grain of the extract of belladonna with from two to five grains of sulphate of quinia every three or four hours.

Should we have reason, from the occurrence of delirium or the beginning of coma, to suspect that emboli were being deposited, we should, without delay, resort to the use of carbonate ammonia and produce full alkalization of the blood as speedily as possible. Such are the general principles of the systemic management of these cases.

What local measures should be employed? The text-books contain a vast variety of remedies to be applied locally. The attempt is made to stop its spread by the use of blisters, nitrate of silver, tincture of iodine, a saturated solution of the sulphate of iron, carbolic acid and a thousand and one other remedies. All this is based upon a fallacy. This condition of the skin is a symptom of the malady and only a symptom. We cannot, as a rule, prevent the spread of the disease by the remedies mentioned. We cannot prevent or limit by such measures the constitutional condition. The simplest local application suffices. I have seen more good from mercurial ointment very much diluted, and from vaseline or lard, than from the most elaborate applications. The strength should be one drachm of mercurial ointment to the ounce of lard or vaseline. If there is reason to fear that the disease will exist as an epidemic, we should, of course, adopt measures to prevent the diffusion of germs. In a simple case like the one before you the proper treatment is that which I have indicated.—*College and Clinical Record.*

---

A TOOTHACHE REMEDY.—Melt white wax or spermaceti, two parts, and when melted add carbolic acid crystals, two parts; stir well till dissolved. While still liquid, immerse thin layers of carbolized absorbent cotton wool, and allow them to dry. When required for use a small piece may be snipped off and slightly warmed, when it can be inserted into the hollow of the tooth, where it will solidify. The ease produced by this simple method is really very great.—*British Medical Journal.*

---

A CASE of death from the inhalation of ether occurred at a clinic at Bellevue Hospital recently. The patient was a boy with apparently sound lungs and heart. He was under ether for about an hour and a half, when he suddenly ceased to breathe, and all efforts at resuscitation failed.—*Medical and Surgical Reporter.*

---

SURGEONS J. S. BILLINGS and J. S. BROWN, U. S. A., have been detailed to attend the International Health Exhibition, which meets at South Kensington in May next, and to represent the Department at the International Medical Congress, which will convene at Copenhagen in August next.

---

THE COURIER-RECORD OF MEDICINE, Fort Worth, Texas, is one of the best journals that comes to our office. Every issue is well filled with original matter. Success to it.

